# Developing and planning country-specific integrated knowledge translation strategies: experiences from the GELA project in Malawi, Nigeria, and South Africa

**DOI:** 10.1186/s12889-024-18934-8

**Published:** 2024-05-27

**Authors:** Bey-Marrié Schmidt, D. Mabetha, M. Chibuzor, G. Kunje, D. Arikpo, E. Aquaisua, S. Lakudzala, N. Mbeye, E. Effa, S. Cooper, T. Kredo

**Affiliations:** 1 Health Systems Research Unit, South African Medical Research Council, Cape Town, South Africa; 2 MRC/Wits Rural Public Health and Health Transitions Research Unit (Agincourt), School of Public Health, Faculty of Health Sciences, University of the Witwatersrand, Johannesburg, South Africa; 3 Cochrane Nigeria, Institute of Tropical Diseases Research and Prevention, University of Calabar Teaching Hospital, Calabar, Nigeria; 4 Evidence Informed Decision Making Center, Department of Community and Environmental Health, School of Global and Public Health, Kamuzu University of Health Sciences, Lilongwe, Malawi; 5 Cochrane South Africa, South African Medical Research Council, Cape Town, South Africa

**Keywords:** Integrated knowledge translation, Stakeholder engagement, Guidelines, New born and child health, Malawi, Nigeria, South Africa

## Abstract

**Background:**

The Global Evidence, Local Adaptation (GELA) project aims to maximise the impact of research on poverty-related diseases by increasing researchers’ and decision-makers’ capacity to use global research to develop locally relevant guidelines for newborn and child health in Malawi, Nigeria and South Africa. To facilitate ongoing collaboration with stakeholders, we adopted an Integrated Knowledge Translation (IKT) approach within GELA. Given limited research on IKT in African settings, we documented our team’s IKT capacity and skills, and process and experiences with developing and implementing IKT in these countries.

**Methods:**

Six IKT champions and a coordinator formed the GELA IKT Working Group. We gathered data on our baseline IKT competencies and processes within GELA, and opportunities, challenges and lessons learned, from April 2022 to March 2023 (Year 1). Data was collected from five two-hour Working Group meetings (notes, presentation slides and video recordings); [2] process documents (flowcharts and templates); and [3] an open-ended questionnaire. Data was analysed using a thematic analysis approach.

**Results:**

Three overarching themes were identified: [1] IKT approach applied within GELA [2], the capacity and motivations of IKT champions, and [3] the experiences with applying the GELA IKT approach in the three countries. IKT champions and country teams adopted an iterative approach to carry out a comprehensive mapping of stakeholders, determine stakeholders’ level of interest in and influence on GELA using the Power-Interest Matrix, and identify realistic indicators for monitoring the country-specific strategies. IKT champions displayed varying capacities, strong motivation, and they engaged in skills development activities. Country teams leveraged existing relationships with their National Ministries of Health to drive responses and participation by other stakeholders, and adopted variable communication modes (e.g. email, phone calls, social media) for optimal engagement. Flexibility in managing competing interests and priorities ensured optimal participation by stakeholders, although the time and resources required by IKT champions were frequently underestimated.

**Conclusions:**

The intentional, systematic, and contextualized IKT approach carried out in the three African countries within GELA, provides important insights for enhancing the implementation, feasibility and effectiveness of other IKT initiatives in Africa and similar low- and middle-income country (LMIC) settings.

## Background

Integrated Knowledge Translation (IKT) research and practice is of increasing interest for funders, to promote the value of research and justify spending on research, and for policy-makers, to show accountability in decision-making and enhance health system performance and population health [1, 2]. IKT is an ongoing relationship between researchers and decision-makers (e.g. patients, health professionals, policy-makers) for the purpose of engaging in a mutually beneficial research project to support evidence-informed decision-making [3, 4]. IKT therefore has the potential to facilitate the co-production of knowledge that is relevant and timely for decision-making and increase the uptake and use of research evidence in health programmes, policies and practice [2]. However, some challenges exist with IKT. These include, negative attitudes and poor knowledge about IKT, limited institutional support and buy-in, limited personnel and financial resources, lack of knowledge and skills and relationships to implement IKT, and scarce research evidence that is relevant and timeous for addressing context-specific policy and practice issues [5–9].

**Fig. 1:**
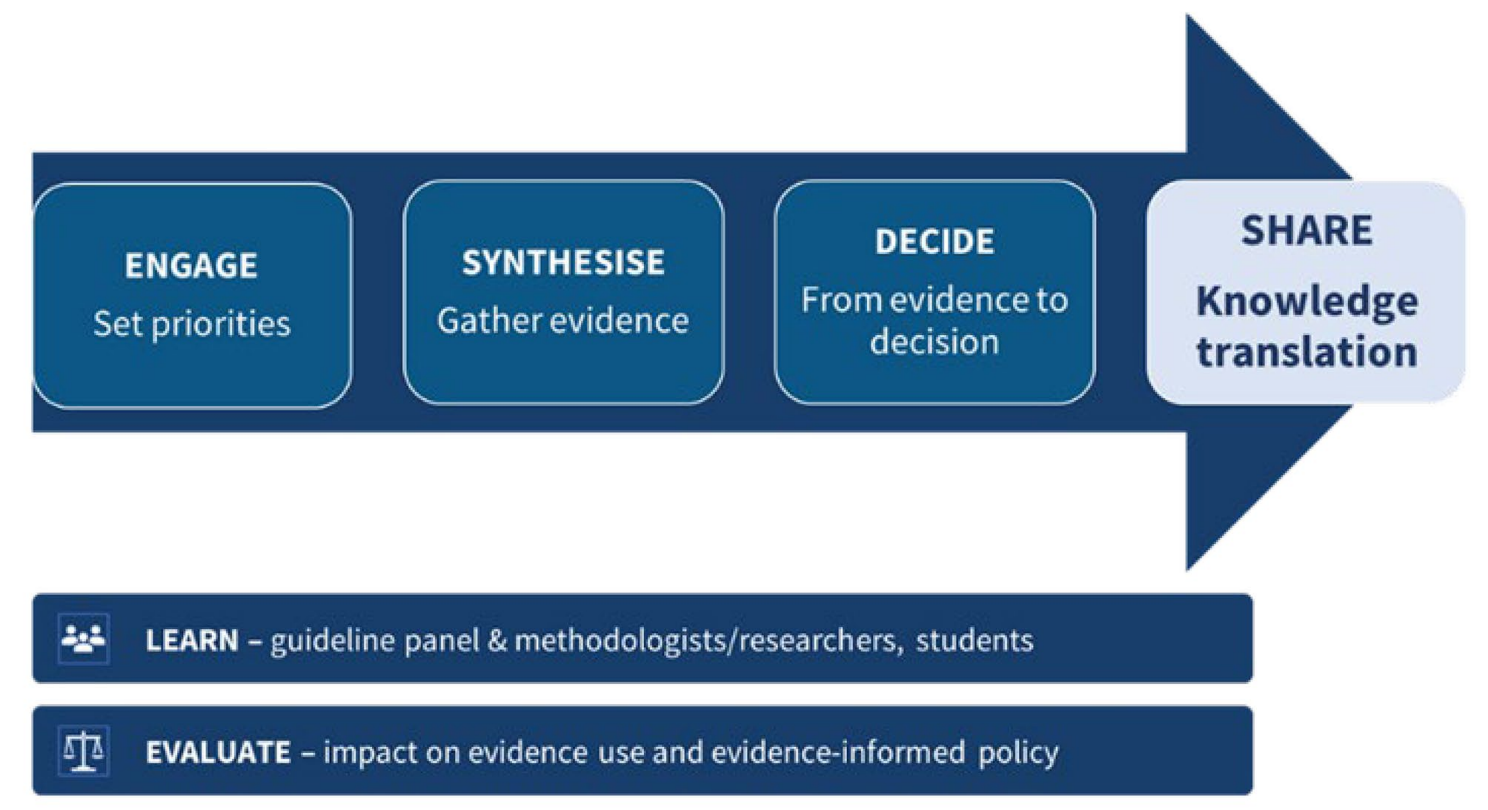
GELA work packages

Despite these challenges, IKT has been embedded in various research projects over the past decade [3, 10], and it is generally assumed that IKT can positively influence research, policy and practice [11]. IKT remains an emerging research field, with additional research needed on IKT theory, the optimisation of IKT processes, and methods for embedding IKT in research and evaluating its value [12–15]. There are particularly limited studies from low- and middle-income countries (LMICs) documenting the development, implementation, monitoring and evaluation of IKT [3, 10, 16]. IKT is an important part of the Global Evidence, Local Adaptation (GELA) project, which aims to produce research and build capacity among researchers and decision-makers for the development and adaptation of guideline recommendations for newborn and young child health in Malawi, Nigeria and South Africa. While the global under-5 mortality rate fell to 37 deaths per 1000 live births in 2020, children in sub-Saharan Africa continued to have the highest rates of mortality in the world at 74 deaths per 1000 live births – 14 times higher than the risk for children in Europe and North America [17]. GELA consists of seven work packages (Fig. 1), including evidence synthesis, guideline recommendation formulation, and IKT. The fourth work package, called “SHARE”, includes the development, implementation, and monitoring and evaluation of IKT strategies.

## Objective

This paper aims to describe the process and experiences with developing and planning for the implementation of country-specific IKT strategies in Malawi, Nigeria and South Africa within the GELA project.

## Methods

**Fig. 2:**
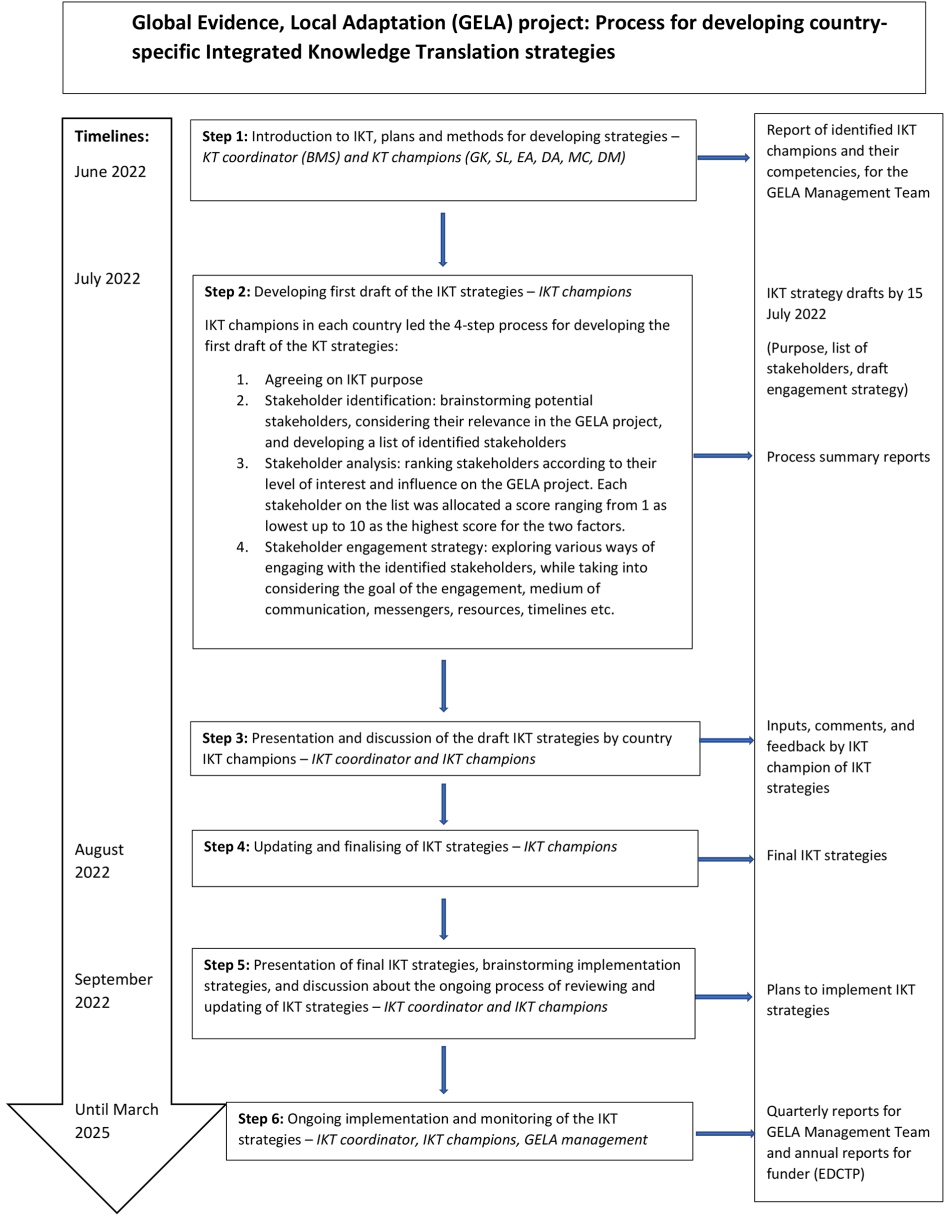
Figure 2

At the start of the project, each country partner identified IKT champions to join the GELA IKT working group. The working group consisted of six IKT champions: two from Malawi (GK, SL), three from Nigeria (MC, DA, EA) and one from South Africa (DM). The champions were responsible for coordinating the development, implementation and monitoring of the country-specific IKT strategies, which outlined the methods for engaging stakeholders relevant to the objectives and outcomes of GELA. In addition, the working group included the GELA work package 4 co-lead (BMS) who served as an IKT coordinator, and was responsible for facilitating collective learning about IKT and assisting with the development, implementation, and monitoring of the country-specific IKT strategies. The IKT working group held five two-hour meetings between April 2022 and March 2023 (GELA Year 1) on Zoom. The meetings provided IKT champions with the opportunity to share updates on progress made with developing and implementing the country-specific IKT strategies, to reflect on any opportunities realised and challenges faced, and to collectively identify solutions for mitigating the identified challenges. Parallel to the meetings, the IKT champions developed the following documents:

- Overall GELA IKT approach (see Fig. 2): a flowchart of the IKT approach taken within GELA, involving the IKT working group, the broader country teams, and the GELA management team.
- Stakeholder map: a list of stakeholders relevant to GELA, identified through brainstorming and snowballing.
- Stakeholder interest and influence matrix: a list of stakeholders relevant to GELA ranked by their presumed interest in and influence on GELA.
- Three country-specific IKT strategies (also known as ‘tracking sheets’): a strategy of the purpose, the medium or forum, messenger, timing and resources for engagement for each of the prioritised stakeholders. Each strategy also included IKT champions’ reflections on engagement activities and processes, such as feedback from stakeholders, lessons learned, opportunities identified, and challenges encountered.

Questionnaire completed by IKT champions: a set of open-ended questions on IKT competencies, challenges, opportunities, processes and lessons from Year 1.

The IKT coordinator (BMS) gathered all the meeting notes, stakeholder maps, and IKT strategies developed by the working group during Year 1 of GELA. Additionally, BMS developed an open-ended questionnaire and disseminated it to the IKT champions via email, including informed consent. BMS and DM jointly drafted a flowchart of the IKT approach implemented within GELA. Following a thematic analysis process, BMS read through all the documents to familiarise herself with information on the process and experiences with developing the country-specific IKT strategies. Thereafter, she highlighted (coded) relevant sections of information with different colours to distinguish between different topics of interest (i.e. process, capacity, motivations, challenges, opportunities, lessons). BMS then systematically went through the topics to formulate preliminary themes. Before finalising the themes, IKT champions (DM, GK, SL, MC, DA, EA) verified the preliminary themes and contributed to the write-up of the themes.

### Findings

The findings are organised into three themes below. The first theme, ‘IKT approach applied within GELA’, consists of five sub-themes, with each sub-theme describing a step in the process for developing the contextualised IKT strategies. The second theme is on ‘Capacity and motivations of IKT champions’. The third theme, ‘Experiences with applying the GELA IKT approach in Malawi, Nigeria and South Africa’ consists of five sub-themes, describing the challenges and opportunities, and the lessons learned by country teams while developing and planning for the implementation of the country-specific IKT strategies.

### Theme 1: IKT approach applied within GELA

The IKT coordinator asked each country team to identify at least one individual to serve as an IKT champion when the GELA project started in April 2022. Six IKT champions were identified among the three countries (Malawi = 2, Nigeria = 3, South Africa = 1). The first IKT working group meeting took place in June 2022. Following this meeting, each country team, coordinated by the IKT champion(s), worked together to develop their country-specific IKT strategy. The process for developing the strategies consisted of five steps: (1) defining the purpose of the IKT strategy, (2) stakeholder identification, (3) stakeholder analysis and mapping, (4) stakeholder engagement plan, and (5) monitoring plan.

### Step 1: defining the purpose of the IKT strategy

The process followed for IKT was similar in all three countries, drawing from training done by Jessani et al. [18] and previous work done by the Collaboration for Evidence-Based Healthcare and Public Health in Africa (CEBHA +) project [19]. The IKT working group used materials from Jessani et al. and the CEBHA + project to clarify what the purpose of IKT is, how to conduct stakeholder mapping and analysis, and why evaluating IKT is important. Each country team initiated the process by discussing the relevance and importance of the GELA IKT approach in their local context. The purpose of the IKT approach was to raise awareness of GELA, engage local stakeholders in GELA activities, and continuously disseminate and communicate GELA activities and outcomes throughout the project period.

### Step 2: stakeholder identification

Each country team started with brainstorming a list of country-specific stakeholders working in the field of child health who are relevant to the GELA project activities and outcomes. Potential stakeholders were identified by leveraging existing relationships, contacting known experts in the child health field, undertaking a desk review of national policy documents on child health, internet searches, and applying the snowballing technique. Country teams then compiled a list of all potential stakeholders. Examples of stakeholders were government departments, national public health agencies, professional health associations, academic and research institutions, non-government organisations, civil society organisations and multilateral agencies (e.g. World Health Organisation and the United Nations Children’s Fund. After several discussions about the potential role of stakeholders and their interest in, and influence on, the success of GELA, country teams prioritised specific stakeholders for ongoing engagement. Prioritising stakeholders was an important step for ensuring that the IKT approach applied within GELA is feasible based on available time and resources.

### Step 3: stakeholder analysis and mapping

A systematic process was employed to rank each of the prioritised stakeholders according to their level of interest in and influence on GELA. Each member within a country team ranked each stakeholder from 1 (lowest level of interest/influence) to 10 (highest level of interest/influence), and an average score was calculated. The average score for each stakeholder was plotted onto an interest-influence matrix consisting of four quadrants (avoiders, silent boosters, blockers, and champions), using a Microsoft Excel template. Level of influence was plotted on the vertical axis (y-axis) and level of interest on the horizontal axis (x-axis). The matrix helped country teams determine how to engage with their prioritised stakeholders (monitor, keep informed, engage and consult, and keep satisfied) and what resources (time, financial and personnel) are required.

### Step 4: stakeholder engagement plan

The previous steps [1–3] were taken to develop country-specific IKT strategies (stakeholder engagement plans). Each stakeholder engagement plan summarised who the relevant stakeholders are to a research project; the purpose for engagement; the messages to be communicated; the modes and formats for engagement; the appropriate messengers; and the time and resources required for engagement. Different stakeholders were included in the stakeholder engagement plan, so step 4 involved country teams deciding which approach is best suited to each individual stakeholder (e.g. inform, consult, involve, collaborate and empower). Since different stakeholders were engaged for different purposes throughout the project, the IKT strategies were continuously updated.

Although all country teams applied the IKT approach to developing their specific strategies, each team reached a consensus on the final IKT strategy in a different way. In South Africa, the IKT strategy was developed by a subgroup of the country team (consisting of the country lead, IKT champion, communications officer and two researchers), and then shared with the broader country team for input and finalisation. In Malawi, the development of the IKT strategy relied on the country lead, the IKT champion, the postdoctoral fellow and the communications officer. In Nigeria, the three IKT champions developed a list of relevant stakeholders, and then shared the list with ten experts in child health affiliated to their institution for validation and ranking, after which they finalised the strategy.

### Step 5: monitoring plan

The final step was about monitoring the implementation of the country-specific IKT strategies. Once the country-specific strategies were developed, the IKT working group collectively decided on the monitoring information to be captured per stakeholder for each goal of engagement in the IKT strategies. The monitoring information included : type of indicator per goal (e.g. reach, usefulness, relationship), feedback from the stakeholder, status of the engagement (goal achieved or not), challenges faced, opportunities identified, and lessons learned during the engagement.

### Theme 2: capacity and motivations of IKT champions

The capacity and motivations of IKT champions were captured in the open-ended questionnaires. All the IKT champions held a Master’s degree in a relevant field (e.g. public health, information science). They all attended additional training in evidence-informed decision-making and two of the six had attended an introductory knowledge translation course. Two of the six IKT champions were newly recruited to work on GELA project, while the other four were already working for their institutions on other projects. They were all employed in other roles on the GELA project, for example in project management and research, so most of them only worked on IKT for approximately one day a week. IKT champions had varied experience in engaging stakeholders as part of a research project; three of the IKT champions had less than one year of experience, two had about five years of experience, and one had more than ten years of experience. IKT champions generally felt confident and motivated to coordinate IKT for their country teams, but they also saw their role as an opportunity to receive further training and share what they had learned previously. Some IKT champions saw their roles as evolving because they were learning new skills on the job (e.g. on monitoring and evaluating IKT), while others saw the implementation of IKT as an opportunity to strengthen relationships with stakeholders they engaged with on previous projects.

Theme 3: Experiences with applying the GELA IKT approach in Malawi, Nigeria and South Africa.

Country teams, comprising IKT champions and other project staff, followed a rigorous process for developing their IKT strategies. Their experiences (challenges and opportunities) with developing and implementing the country-specific IKT strategies and the lessons they learned in the first year of GELA are described below.

#### Leveraging existing relationships

Country teams were able to identify relevant stakeholders through brainstorming and existing networks. One challenge they faced was to identify individuals representing prioritised stakeholders and to obtain their functional email addresses and telephone numbers. However, this challenge was overcome by leveraging existing relationships with stakeholders, contacting institutional receptions, and searching the internet. The teams had existing relationships with their national Ministries of Health (MoH), so they used those relationships to identify and connect with other stakeholders. The Nigeria team, specifically, were able to quickly connect with child health experts based at their institution to obtain priority topics and suggestions of additional stakeholders for the steering group.

#### Communicating with stakeholders

Another challenge country teams faced was related to identifying the right communication channels for prioritised stakeholders. Project leads first reached out to stakeholders to introduce GELA and project staff via email. Stakeholders were asked to acknowledge the introductory emails, but despite a few follow-ups, most stakeholders did not respond. IKT champions then contacted stakeholders who did not respond to emails via telephone calls, to introduce themselves, gauge interest in GELA and ask about appropriate communication channels. Even in instances where IKT champions got positive responses from stakeholders via telephone calls, stakeholders did not necessarily become more responsive in future communication. Country teams learnt that using various communication channels improved the response rate. Even when stakeholders said that they preferred email communication, teams followed up and shared reminders via telephone calls. Often this provided stakeholders with an opportunity to clarify the email content and to provide context to answers before they responded to emails. The Malawi team, specifically, learned that they could not implement some of their stakeholders’ communication preferences. For example, most of their stakeholders preferred in-person meetings, but they held most meetings with stakeholders via video conferencing platforms, such as Zoom and Microsoft Teams, because of logistical issues and financial constraints.

Additionally, country teams were challenged with delayed or lack of responses from stakeholders, as it slowed down engagement processes, which in turn affected the start of some project activities. For example, the processes for mapping country-specific stakeholders during the development of the IKT strategies also helped teams identify stakeholders who could be invited to the steering groups, but delayed responses from invited stakeholders led to the late start of the steering groups. To reduce the response time, the Malawi team engaged stakeholders via text messages, e.g. using WhatsApp, to familiarise them with GELA and what participation in the steering group would entail.

#### Using appropriate messengers

In addition to using various communication channels to reach stakeholders, country teams also sought help from appropriate ‘messengers’. One challenge that the Nigeria team faced was identifying relevant stakeholders to invite to their steering group. They engaged the Federal Ministry of Health (FMOH) about this problem, and a FMOH staff member contacted representatives of multilateral agencies and civil society organisations that they already had relationships with, to connect them with the Nigeria team. Further, the Nigeria team invited other stakeholders to the steering group via appointment letters signed and disseminated by a FMOH staff member. These processes required extensive follow-up with the FMOH staff member, given their competing work responsibilities.

Similarly, the Malawi team also sought the help of government ministries in reaching relevant stakeholders across different sectors. Engaging the MoH as the key ‘messenger’ proved more effective than the country team sending out emails themselves. For example, when the country team sent out invitations for stakeholders to join the steering group and to attend an orientation meeting, only three stakeholders responded to the email and attended the meeting. However, when the country team wanted to recruit stakeholders to the guideline development panel, they asked the MoH to send out the invitations. All those who were invited via the MoH to the orientation meeting responded to the invitation and attended the meeting. Country teams learned that working with key messengers, like the MoH, is important for implementing IKT. Developing strong relationships with such messengers helped them get connected to new stakeholders and identify those stakeholders already working on child health guidelines (which was a key outcome of GELA).

For the South Africa team, including cc’ing the principal investigator in emails and mentioning her institutional position in emails to stakeholders led to more favourable responses. The country team learned that successful stakeholder engagement sometimes relies on the messenger’s function, e.g. role in the project, institutional position, and prior contact with a stakeholder. Subsequent follow-ups to emails by the IKT champion also led to most stakeholders responding.

#### Managing competing priorities and interests

It was also challenging for country teams to deal with the competing priorities and interests of stakeholders. For example, in Malawi, a few stakeholders hesitated to share information about other relevant stakeholders with the country team, out of concern that their own priorities and interests would be neglected if too many stakeholders were involved in GELA. As such, the Malawi team initially spent a lot of time managing expectations amongst known stakeholders and trying to identify new stakeholders. They also learned to get input from a variety of relevant stakeholders who had different levels of interest in, and influence on, GELA, so that they could know about and manage different expectations. It was also challenging for country teams when some stakeholders who were invited to participate in the steering group were unable to attend meetings consistently because of competing work priorities.

#### Managing internal administrative processes

At the start of the project, country leads were faced with the challenge of getting funds to their institutions timeously, initiating recruitment, and finalising staff contracts. The delay in these administrative processes resulted in a slow start to the IKT work, as recruitment of some team members was still underway and funds were not available for engagement activities (e.g. airtime to call stakeholders and transport to meet with them). New project staff also needed to be orientated to GELA, get an introduction to IKT, and organise themselves administratively so that they can engage stakeholders. The South Africa team, specifically, experienced challenges with identifying a convenient meeting time for all team members (across the two South African institutions involved in GELA). As such, not all team members were able to attend meetings to discuss and reach consensus regarding identified stakeholders. This delayed the process of reaching out to stakeholders as team members needed time to go through meeting minutes before providing their inputs. However, once all administrative and financial constraints were addressed, the country teams were able to plan for IKT properly. The working group meetings also helped IKT champions learn more about IKT and brainstorm solutions for stakeholder engagement. They also learned about the time and resources required to implement IKT and how to account for this in future grant proposals, especially considering different stakeholders’ needs and preferences around engagement.

## Discussion

### Summary of the findings

This paper has described the process and experiences with developing and planning for the implementation of country-specific IKT strategies in Malawi, Nigeria and South Africa. Drawing on IKT approaches from the CEBHA + project [3, 10, 13], country teams, with the guidance of IKT champions, developed and planned the implementation of country-specific IKT strategies (see Fig. 2). IKT champions had varying capacity, motivations and experience with IKT, but they generally felt confident and saw their roles as an opportunity to learn new skills and establish or strengthen relationships with stakeholders. The IKT work carried out in the first year of GELA highlighted some challenges, opportunities and lessons. Examples include leveraging existing relationships and using appropriate messengers to identify stakeholders, get their contact details, and reach out to them. Additionally, country teams were also concerned with managing stakeholders’ competing priorities and interests, and setting up internal administrative processes needed for the IKT work. The intentional and systematic IKT approach carried out within GELA, specifically contextualised for the three African countries, adds to the limited numbers of studies that describe the practical application of IKT in LMIC settings [10].

### Limited literature describing and evaluating IKT

There has been an increase in the number of studies on IKT since the last few years [3, 16–18]. However, few studies describe the development, implementation, monitoring and evaluation of IKT strategies embedded in public health projects in LMICs, specifically. A recent study by Sell et al. (2023) described the development, implementation and monitoring of site-specific IKT strategies across Ethiopia, Malawi, Rwanda, South Africa and Uganda. Similar to this study, Sell et al. found that tailoring the overall IKT approach according to context- and stakeholder-specific needs and preferences was highly beneficial. In their study, it led to the inclusion of some atypical IKT stakeholders, greater responsiveness during engagement, balancing of existing and new strategic partnerships, and an enhanced understanding of different research contexts [10]. A study by Jessani et al. (2021) described the ‘messy’ phases of developing, implementing and montoring an IKT approach in South Africa. Similar to this study, Jessani et al. described the challenges of IKT in complex and continously changing contexts. They also advocated for an adaptive IKT approach, making IKT researchers agile, responsive, relevant, and useful in supporting key decision-makers deliberating policies and practices [3]. This study adds to the literature by describing practical challenges, opportunities, and lessons learned from devloping and planning the implementation of three IKT strategies.

### Key lessons for IKT researchers

The first lesson is the necessity of working around the *‘messiness’ of IKT* through a non-linear and iterative approach. The six steps of the GELA IKT approach were conceptualised as linear (see Fig. 2), however in practice, the approach needed to be adaptive and flexible. For example, step 2 was adapted for each country team; the Nigeria country team developed their IKT strategy in a linear manner (i.e. purpose, stakeholder identification, stakeholder analysis, strategy), while the South Africa team developed their IKT strategy intermittently. Country teams repeated steps 2 and 3 a few times before achieving steps 4, and step 4 was continuously repeated as the project activities progressed.

The second lesson is the importance of *contexualising IKT*. The IKT working group developed a standarised template for presenting the IKT strategies, but the contents of the three IKT strategies differed. IKT strategies were tailored; for example, according to stakeholder preferences, availability of resources, appropriate messengers and project objectives and timelines. Additionally, IKT strategies were tailored according to the extent of engagement; stakeholders who were engaged frequently and across different project activities (e.g. priority setting, steering groups, guideline panels) versus stakeholders who were engaged intermittently throughout the project duration.

And the third lesson is not underestimating the amount of *time and resources required for IKT*. Developing and planning the implementation of the IKT strategies included maintaining and establishing relationships, tailoring communication, involving messengers, managing competing priorities and interests, and setting up internal IKT and administrative infrastructure. The IKT working group learned that engagement activities require time, personnel and resources.

### Limitations of the study

This paper only described the experiences of the IKT champions, and not those of other relevant stakeholders, for the first year of the GELA project. We do, however, aim to report on stakeholders’ experiences in future publications when we report on the full implementation and evaluation of the IKT strategies. Although we used the IKT strategies (‘tracking sheet’) and meeting minutes to capture our reflections, some reflections occurred on an adhoc basis (e.g. in non-IKT related meetings). This may have resulted in missing data and limited the analysis or potential for further elaboration.

## Conclusion

While there is increased recognition that IKT can positively influence research, policy and practice, knowledge about the development, implementation and evaluation of IKT in LMICs is scarce. In this paper we have contributed to redressing this knowledge gap by highlighting the challenges and opportunities experienced with developing and planning the implementation of country-specific IKT strategies in Malawi, Nigeria and South Africa for the Global Evidence, Local Adaptation (GELA) project. Our experiences reflect and build on the findings of the few other studies exploring IKT strategies embedded in public health projects in LMICs. The initial lessons we have learnt – the importance of taking a non-linear and iterative approach, of contexualising IKT, and of being cognizant of the time and resources required for IKT – provide important insights for helping to enhance the feasibility and effectiveness of other IKT initiatives in Africa and potentially elsewhere. Future research could focus on exploring the effectiveness and sustainability of context-specific IKT strategies in various LMIC contexts and settings. Longitudinal studies could assess the impact of IKT on health outcomes and health systems strengthening in LMICs, while comparative studies could identify best practices and lessons learned for future IKT approaches.

## Data Availability

The datasets generated and/or analysed during the current study are not publicly available due to the small number of responses and potentially identifiable information. Subsets of the datasets may be made available from the corresponding author on reasonable request.

## Abbreviations

FMOH: Federal Ministry of Health
GELA: Global Evidence, Local Adaptation
IKT: Integrated Knowledge Translation
LMIC: Low- and middle-income country
LMICs: Low- and middle-income countries
